# LCP 140° Pediatric Hip Plate for fixation of proximal femoral valgisation osteotomy

**DOI:** 10.1007/s11832-014-0550-y

**Published:** 2014-01-28

**Authors:** Claudia C. Sidler-Maier, Kerstin Reidy, Hanspeter Huber, Stefan Dierauer, Leonhard E. Ramseier

**Affiliations:** Orthopaedic Division of the Department of Surgery, University Children’s Hospital, Steinwiesstr. 75, 8032 Zurich, Switzerland

**Keywords:** Femoral osteotomy, Valgisation, Pediatric Hip Plate

## Abstract

**Purpose:**

Femoral osteotomy is one of the most widely performed reconstructive operations in pediatric orthopedic surgery. Many implants for fixation have been used, but so far there is no literature about the application and outcome of the LCP 140° Pediatric Hip Plate for proximal femoral valgisation in children.

**Methods:**

Data of patients with a valgisation of the proximal femur using the LCP 140° Pediatric Hip Plate between February 2011 and July 2012 were retrospectively collected and analyzed.

**Results:**

We included 10 patients (11 hips) with a mean follow-up of 15.3 ± 6.3 months (range 5.6–23 months). The mean age was 9.6 ± 1.2 years (range 7.3–11.8 years) with a mean hospital stay of 5.2 ± 1.7 days (range 3–9 days). Callus formation was observed in all cases at 6 weeks postoperative control and consolidation was shown after a mean time of 14.1 ± 2.3 weeks (range 12.1–19.1 weeks). There was no delayed union or any case of non-union in our series. The stability of the operative reduction including the corrected neck-shaft angle (mean 19° ± 7.9°; range 10.5°–38.5°) was maintained during the follow-up period. No cases of recurrence (varisation) or complications requiring further treatment or revision were observed.

**Conclusions:**

In our series, the 140° LCP Pediatric Hip Plate was shown to be safe and applicable in the clinical setting with good results. We therefore consider this device to be valuable for the correction of pathologic varus conditions of the proximal femur in children.

## Introduction

Femoral osteotomy is one of the most widely performed reconstructive operations in pediatric orthopedic surgery [1–3, 21], with applications in a variety of congenital disorders such as developmental dysplasia of the hip, congenital coxa vara, and neuromuscular diseases including cerebral palsy (CP) and spina bifida, but also in acquired conditions including Perthes’ disease, slipped capital femoral epiphysis, and deformity after infection or traumatic injury [3, 11–13, 21–24]. Many different implants have been used for the fixation of proximal femoral valgisation osteotomies [2–4, 11–13, 15, 16, 18, 20–25] with variable success and high recurrence rates [17, 19, 20]. However, none of the reported techniques appeared to be superior, and complication rates up to 42 % have been described [1, 3, 4, 13, 15, 17, 18]. In the literature, inter- and sub-trochanteric osteotomies are associated with a variety of problems, such as prominent hardware, infections, hematoma, chisel malposition, femoral neck fractures, loss of fixation or correction, heterotopic ossification, plate breakage, or avascular necrosis of the femoral head, especially when using angular blade plates [2–4, 13].

An established fixation system, the proximal femoral locking compression plate (LCP) for pediatric hips, which has a low lateral profile system, has been developed to make these operations safer and less demanding [4]. The use of locking screws aims at a stable fixation even in osteoporotic bone, and should therefore reach and maintain a more precise angular reduction without loosening or cutting out [2, 4, 5]. In addition to the enabled early postoperative weight bearing based on the biomechanical properties [4, 8], the risk of disturbances to periosteal circulation is minimized [4, 7, 8]. This is the case even in patients with poor bone quality [5–7] due to the low bone-plate contact while using locking screws.

In addition to studies about proximal femoral valgisation osteotomies with other fixation systems, literature is available about the use of the LCP Pediatric Hip Plates for varisation [5] and for valgisation osteotomies of different degrees [2, 4]. The application and outcome of the new valgisation 140° LCP Pediatric Hip Plate has never been investigated nor described exclusively, so far. This device has been used since 2011 in our institution.

The aim of this study was therefore to describe the clinical results using the 140° LCP Pediatric Hip Plate for proximal femoral valgisation osteotomy, to evaluate its applicability and reliability in congenital and acquired disorders with regard to perioperative details, radiographic results, and the eventual complications.

## Patients and methods

We reviewed the clinical records and radiographs of all patients who underwent a proximal femoral valgisation osteotomy between February 2011 and July 2012. Retrospective analysis included chart review with an assessment of gender, age, BMI, diagnosis, preoperative and postoperative imaging, intraoperative handling, noting of additional operations, complications, length of hospital stay, correction of the planned neck-shaft angle (NSA), limb-length discrepancy (LLD), consolidation rate, and time to consolidation of the osteotomy as well as the follow-up time.

Our population was heterogenic regarding the underlying disease (Legg-Calvé-Perthes disease: six patients, congenital femoral defect: two, necrosis after pathological fracture: one child, and coxa magna in the context of Smith-McCort-Syndrome: one patient), as shown in Table 1.

**Table 1:** Diagnosis and demographics of the patients treated operatively with the 140° LCP Pediatric Hip Plate

ID	Age at OP (years)	Gender	BMI (kg/m^2^)	Diagnosis	Side	Mobility
1	7	Boy	11.6	Congenital femoral defect	R	Walker
2	9	Boy	19.6	Legg-Calvé-Perthes disease	L	Walker
3	8	Boy	13.9	Legg-Calvé-Perthes disease	R	Walker
4	9	Boy	28.5	Legg-Calvé-Perthes disease	L	Walker
5	9	Boy	27.9	Coxa magna in the context of Smith-McCort-Syndrome	R/L	Walker
6	11	Boy	15.8	Legg-Calvé-Perthes disease	R	Walker
7	10	Boy	22.7	Legg-Calvé-Perthes disease	R	Walker
8	9	Girl	18.7	Congenital femoral defect	R	Walker
9	10	Girl	21.0	Necrosis of femoral head after pathological femoral neck fracture (bone cyst)	R	Walker
10	9	Boy	16.2	Legg-Calvé-Perthes disease	L	Walker

*BMI* body mass index, *R* right, *L* left

All parents or legal representatives of the patients gave written consent to the operation.

### Surgical technique

The planning as well as the procedure was performed according to the technique guide provided by Synthes^®^, Switzerland [10].

Preoperatively, each patient received an antibiotic prophylaxis with Kefzol^®^ (Cefazolin, 1st generation cephalosporine).

### Follow-up

Anteroposterior (AP) pelvic radiographs and axial X-rays (Lauenstein) were realized preoperatively, as well as 6 and 12 weeks postoperatively, and the measurements were taken by two different experienced pediatric orthopedic surgeons. During the follow-up period for each of the 10 patients, an orthoradiograph was performed: the patient was standing on both feet (if necessary in his orthosis) with the patellae centered on the knee joints in order to standardize the imaging (internal/external rotation of the femur). On these radiographs, the limb length was measured radiologically from the middle of the acetabulum caudally downwards to the middle of the ankle.

The postoperative neck-shaft angle was considered to be as planned if the difference to the preoperatively planned one was ±10°, assuming that in the two different measurements of each case, the individual error of measurement might be 5°. This was based on Wilson’s [14] study about intra- and interobserver reliabilities where an interobserver reliability in measuring neck-shaft angles of ±6° was reported. We therefore consider a 5° possible error in each measurement as reasonable (2 × 5° = 10° for both measurements).

According to Keating et al. [9], we defined radiologic union as evidence of bridging of three of the four cortices on standard ap and lateral radiographs.

All hardware was routinely removed.

## Results

### Patients demographics

Our study included 10 patients (11 operated femurs: four left and seven right femurs), among them one patient with a bilateral operation. The mean age at operation of all the children (eight boys, two girls) was 9.6 ± 1.2 years (range 7.3–11.8 years). The mean weight of our patients was 36 ± 9.6 kg (range 16.8–51.2 kg) with a mean BMI of 20.4 ± 5.9 kg/m^2^ (range 11.6–28.5 kg/m^2^). Three patients were obese, presenting with a BMI of about 28 kg/m^2^. Before and after the operation all children were ambulatory (Table 1).

The mean follow-up time was 1.7 ± 0.5 years with a range of 1–2.5 years.

### Surgery and postoperative handling

In seven cases, a 3.5 140° LCP Pediatric Hip Plate was implanted, in four cases we used a 5.0 LCP plate. An additional internal rotation was realized in seven femurs, while there was no rotational correction in the other four cases. Three presented with an additional extension, one with a flexion, and seven osteotomies were performed without any correction in this plane. Two children with congenital femoral defects had an additional surgery at the same time, such as a pelvis reconstruction (1× Dega and 1× Pemberton Osteotomy), and a subsequent Triple-Osteotomy was executed in two other patients with Legg-Calve-Perthes disease. With all patients having had an uneventful surgery, their mean hospital stay was 5.2 ± 1.7 days (range 3–9 days). In four cases, no weight bearing was allowed postoperatively (two patients with a congenital femoral defect, one patient with Perthes’ disease, and one patient with necrosis of the femoral head); four patients were partial-weight bearing (all presenting with Perthes’ disease), and the remaining three were full-weight bearing.

### Radiologic evaluation

**Fig. 1:**
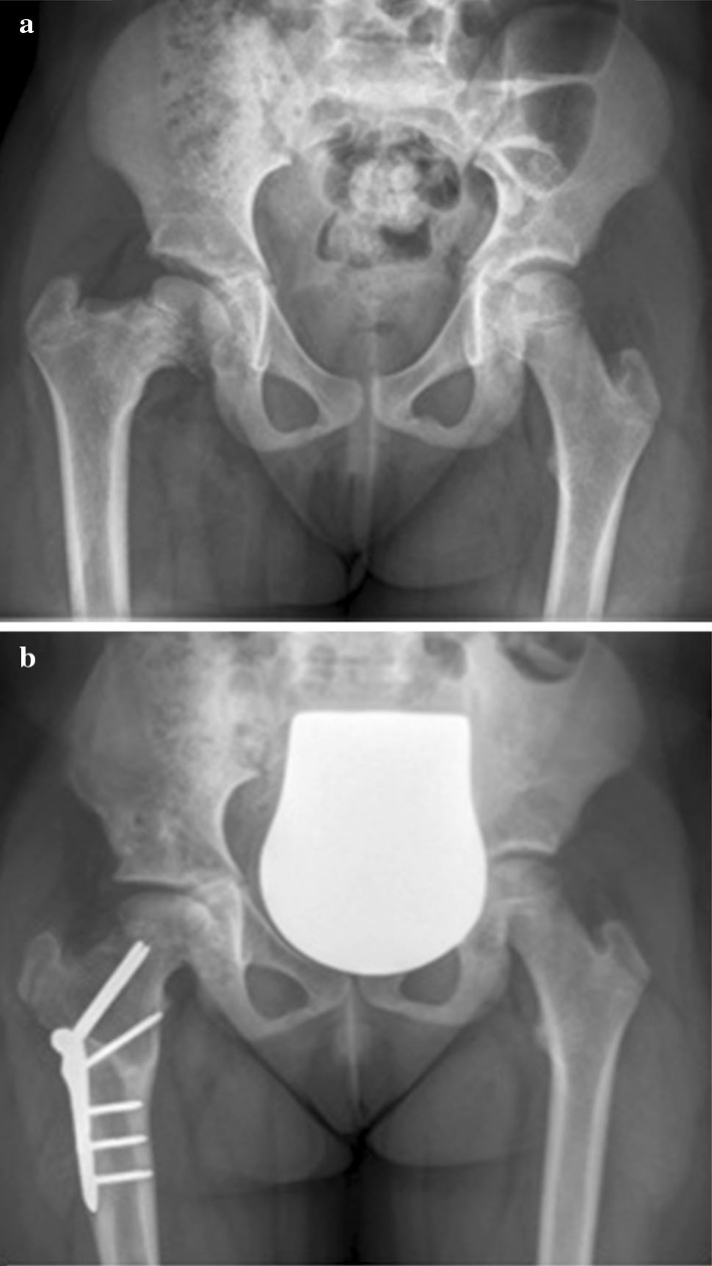
**a** Preoperative X-ray of a 10.5-year old girl presenting with congenital femoral defect. **b** Good reduction is achieved after femoral valgisation osteotomy (30°), and at 1.2 years of follow-up, reduction is maintained with good head coverage

The average preoperative neck-shaft angle was 130° in our series (range 108°–141.5°). Six weeks postoperatively, the mean angle was 149° (average 132°–164°) with a mean corrected neck-shaft angle of 19° ± 7.9° (range 10.5°–38.5°) (Table 2). The postoperative pelvis AP radiographs corresponded with the preoperative planned corrections (mean 25.5°, range 15°–30°) in 9/11 cases according to our defined total possible error of ±10°. In the other two patients, the lack of correction was 15.5° and 16.5°. The mean lack of correction in all 11 cases was 6.5° ± 6.8°, with a range of 8.5°–16.5°. The mean change of the neck-shaft angle during the follow-up period was −3.4° ± 4.3° (range −11° to +2.5°), showing an average neck-shaft angle of 145.6° ± 7.3° (range 130.5°–155.5°) at last radiologic control. Figure 1 shows a preoperative and postoperative radiologic example using a 140° Pediatric Hip Plate. There were no recurrences or complications requiring further treatment or revision, either in the children with a normal BMI or in the obese patients.

**Table 2:** Radiologic outcome

ID	NSA (in degrees)				Mean LLD (in mm)
	Preoperatively	Planed correction	Rx pelvis 6 weeks postoperatively	Last control 5.6–23 months postoperatively	Orthoradiograph 5–22 months postoperatively
1	120.5	20	132	128	44
2	141.5	30	164	165	3
3	139	30	153.5	153	3
4	136	30	149.5	147	1
5	126	30	148	148	12.5
5	140	15	150.5	152	12.5
6	133	25	157.5	157	1
7	128.5	25	146	149	3
8	108	30	146.5	140	25.5
9	131.5	20	148	149	12
10	127	25	144	143	1.5

*NSA* neck-shaft angle*, Rx* X-rays, *LLD* limb-length discrepancy

In our series, callus formation was observed in all cases at 6 weeks postoperative control, and consolidation was shown after a mean time of 3.3 ± 0.5 months (range 2.8–4.5 months). We did not see any delayed unions or any case of non-union.

The mean limb-length discrepancy measured on the orthoradiograph 5–22 months postoperatively by both raters was 10.9 ± 13.3 mm (range 1–44 mm).

In 11 cases, the hardware had been removed at last control with a mean postoperative follow-up time of 1.1 ± 0.3 years (range 0.7–1.6 years) after proximal femoral valgisation osteotomy, and a mean follow-up time after plate removal of 0.6 ± 0.5 years (range 0–1.6 years).

## Discussion

The objective of this study was to critically analyze the application of the 140° valgisation LCP Pediatric Hip Plate, as its use and outcome have never been investigated and described exclusively, to date. In addition to the literature about valgisation osteotomies using other implants or fixation systems [1–3, 11–13, 15–25], there exist only two studies so far that describe the results of valgisation osteotomies with an LCP Hip Plate in children [2, 4]. Joeris et al. [4] performed eight valgisations (out of 30 hips) with an LCP Pediatric Hip Plate of 150° and Khouri et al. [2] carried out nine valgisations (of 59 interventions in total) using LCP valgisation plates without mentioning the exact degree, except in three cases where they used a 120° LCP. However, no studies have dealt only with the 140° valgisation Pediatric Hip Plate.

The surgical technique in our study is as described by the manufacturer Synthes^®^, Switzerland [10], and is similar to the one described by Joeris et al. [4] and Khouri et al [2].

According to Günther et al. [12], deformities of the infantile hip requiring valgisation are encountered very infrequently. Likewise, the number of patients in our study being treated with a valgisation osteotomy is rather little, but larger or at least as big as in others studies [2–4, 15, 19, 22, 24]. Concerning the age at surgery, our population was comparable to the one described in most of the literature on valgisation osteotomies [4, 13, 15, 17, 19, 23–25]. Joeris et al. [4], who used Pediatric Hip Plates of 150°, had more girls and heavier children in their study than we did and presented a population that was even more heterogeneous than ours. Similarly, a comparable heterogeneity was found in studies by Skaggs et al. [22], Burns et al. [15], Günther’s [12], Widman’s [18], and Cordes et al [24].

In general, the applicability and handling of the 140° LCP Pediatric Hip Plate was excellent in our study as well as in Joeris et al.'s [4] LCP Pediatric Hip series. The correction achieved with our patients is similar to the correction reported in other series with different fixations systems. The mean corrected neck-shaft angle in our series was 19° ± 7.9 (range 10.5°–38.5°). We achieved the planned correction in all but two cases, whereas it was successful in 7/8 valgisation osteotomies for Joeris et al [4]. Contrary to us, they did not provide a definition for the achievement of planned correction of the neck-shaft angles, nor did any other author.

However, despite improvement in surgical technique, loss of correction in the proximal femur is common after valgus osteotomy [16] and the recurrence rate has been reported to vary between 10 and 75 % [12, 15–18, 20, 24, 25]. See Table 3 for details. The high recurrence proportion could be explained by the biomechanics of the underlying disorder [16], probably due to incomplete correction and/or loss of fixation [15]. According to Wilson [14], who showed a poor interobserver reliability as mentioned above, the apparent loss of correction in our series is not significant enough to consider it a real recurrence. We only had a mean change of −3.5° ± 4.3° (range −11° to 2.5°) during our follow-up period, which seems far better than what other studies reported [12, 24]. Furthermore, the valgisation correction osteotomies with the LCP 140° Pediatric Hip Plate showed to be stable even in our three obese children (with BMI of 28 kg/m^2^). This new fixation system additionally leads to similar results regarding the correction of the neck-shaft angle, as well as the low complication rate and consolidation time of the osteotomy.

**Table 3:** Comparison between different proximal femoral valgisation osteotomies in the literature

Author	Time to bone union in months (range)	Mean F/u in years (range)	Mean NSA in degrees (range)			Mean loss of correction (degree)	Recurrence	Complications
			Preoperative	Postoperative	Last control			
Desai et al. [17]	4.5 (2–8)	20 (6.2–41.4)	NR	NR	NR	NR	15 %	41.7 %
Yang et al. [24]	NR	4.2 (2–8.3)	95 (65–132)	137 (126–156)	125 (64–152)	15.5	75 %	NR
Cordes et al. [25]	NR	11	NR	NR	NR	NR	NR	NR
Widmann et al. [18]	NR	4.5 (0.8–8.5)	93.5 (70–118)	148.4 (130–165)	129.5 (85–156)	NR	29 %	30 %
Burns et al. [15]	NR	3.2 (1–7.9)	NR	NR	NR	NR	16.7 %	16.7 %
Günther et al. [12]	NR	6.2 (0.8–12.8)	98	156	144	12°	10 %	NR
Skaggs et al. [22]	NR	2.9	NR	NR	NR	NR	None	None
Hau et al. [3]	3 (all but one case)	all: 2.3; minimum: 1	NR	NR	NR	None	None	9 %
Myers et al. [11]	NR	6.5 (5.1–7.9)	NR	NR	NR	NR	NR	NR
Fassier [13]	3	4.3 (1.7–8.1)	84.6 (46–118)	119.5 (106–138)	114.4 (65–133)	NR	NR	12 %
Carrol et al. [16]	NR	7.2	NR	NR	NR	NR	50 %	NR
Sabharwal et al. [19]	NR	2.1 (0.35–3.8)	86 (67–105)	137 (122–141)	NR	None	None	NR
Weighill et al. [20]	NR	7.7 (1–36)	NR	NR	NR	NR	21.9 %	NR
Raney et al. [23]	NR	5.2 (4.5–6.2)	140 (120–161)	153 (130–170)	NR	None	None	NR
Other LCP Hip Plates								
Joeris et al. [4]	1.5–2	NR	NR	NR	NR	NR	NR	16.7 %
Khouri et al. [2]	3	NR	105 (85–105)	121.7 (110–125)	124 (110–150)	None	None	None
This paper	3.3 (2.8–4.5)	1.7 (1–2.5)	130 (108–141.5)	149 (132–164)	145.6 (130.5–155.5)	−3.4 (−11 to +2.5)	None	None

*F/u* follow-up time, *NSA* neck-shaft angle, *NR* not reported

The time until formation of a callus as well as consolidation in our series was comparable to other studies (see Table 3). We did not see any delayed unions or any case of non-union in our series. Even if osteotomies are generally expected to heal well if performed at a young age, this was not the case in other studies with a comparable age at surgery of the patients having valgisation osteotomies. Joeris et al. [4] had 1/8 nonunions in their series, Hau et al. [3] had 1/11, and Burns et al. [15] had 1/12 hips result in non-union.

We did not observe any major complications requiring further treatment or revision, nor did Khouri et al [2]. Other authors have reported complication rates between 9 and 42 % [3, 4, 13, 15, 17, 18].

According to the literature, medial displacement of the femoral shaft during valgisation procedures is associated with a certain amount of secondary postoperative genu valgum [12] as the mechanical axis is moved farther laterally. This can cause a probable occult genu valgum to become clinically apparent [26]. Unfortunately, in our study, preoperative data on the mechanical axis were incomplete, rendering analysis of the surgical results on the medialization effect impossible.

Clinical or radiological limb-length discrepancy was only documented in some studies [17, 23, 24] with a lack of information about the preoperative limb length. As this was also the case in our study, it is difficult to analyze our results or compare it to other literature.

In comparison to other literature about fixation systems for valgisation osteotomies. our follow-up period seems to be rather short (mean 1.7 years, range 1–2.5 years), but our purpose was to present preliminary results of the 140° LCP Pediatric Hip Plate. Besides, neither of the other two authors presenting their outcomes of using LCP Hip Plates mentioned their follow-up time.

The drawback of our study is the retrospective, non-comparative design with a small sample size and short follow-up. Furthermore, our preoperative data concerning the limb-length discrepancy as well as the mechanical axis deviation is incomplete, rendering the analysis of the outcome in this regard impossible.

Nevertheless, this initial reported experience encourages us to believe that the 140° LCP Pediatric Hip Plate is a valuable device by which valgisation correction osteotomies of the proximal femur can be surgically treated in a safe way. Larger randomized series with long-term follow-up should be carried out to compare the 140° LCP Pediatric Hip Plate to other devices in order to determine the long-term outcome of this technique (such as recurrence after years) and to better quantify the risk of clinically relevant complications.

## References

[1] Beauchesne R, Miller F, Moseley C (1992). Proximal femoral osteotomy using the AO fixed-angle blade plate. J Ped Orthop.

[2] Khouri N, Khalife R, Desailly E, Thevenin-Lemoine C, Damsin JP (2010). Proximal femoral osteotomy in neurologic pediatric hips using the locking compression plate. J Pediatr Orthop.

[3] Hau R, Dickens DRV, Nattrass GR, O’Sullivan M, Torode IP, Graham HK (2000). Which implant for proximal femoral osteotomy in children? A comparison of the AO (ASIF) 90 degree fixed-angle blade plate and the Richards intermediate hip screw. J Pediatr Orthop.

[4] Joeris A, Audigé L, Ziebarth K (2012). The locking compression paediatric hip plate: technical guide and critical analysis. Int Orthop.

[5] Rutz E, Brunner R (2010). The pediatric LCP hip plate for fixation of proximal femoral osteotomy in cerebral palsy and severe osteoporosis. J Pediatr Orthop.

[6] Schütz M, Südkamp NP (2003). Revolution in plate osteosynthesis: new internal fixator systems. J Orthop Sci.

[7] Haefeli M, Huber HP, Dierauer S, Ramseier LE (2010). Fixation of subtrochanteric extending/derotational femoral osteotomies with the locking compression plate in ambulatory neuro-orthopaedic patients. J Child Orthop.

[8] Huber H, Haefeli M, Dierauer S, Ramseier LE (2009). Treatment of reduced femoral antetorsion by subtrochanteric rotational osteotomy. Acta Orthop Belg.

[9] Keating JF, OBrien PJ, Blachut PA, Meek RN, Broekhuyse HM (1997). Locking intramedullary nailing with and without reaming for open fractures of the tibial shaft. J Bone Joint Surg Am Mar.

[10] http://www.synthes.com/MediaBin/International%20DATA/036.001.057.pdf. Accessed 25 Apr 2013

[11] Myers GJC, Mathur K, O’Hara J (2008). Valgus osteotomy A solution for late presentation of hinge abduction in Legg-Calvé-Perthes disease. J Pediatr Orthop.

[12] Günther CMJ, Komm M, Jansson V, Heimkes B (2013). Midterm results after subtrochanteric end-to-side valgization osteotomy in severe infantile coxa vara. J Pediatr Orthop.

[13] Fassier F, Sardar Z, Aarabi M, Odent T, Haque T, Hamdy R (2008). Results and complications of a surgical technique for correction of coxa vara in children with osteopenic bones. J Pediatr Orthop.

[14] Wilson JD, Eardley W, Odak S, Jennings A (2011). To what degree is digital imaging reliable? Validation of femoral neck shaft angle measurement in the era of picture archiving and communication systems. Br J Radiol.

[15] Burns KA, Stevens PM (2001). Coxa vara: another option for fixation. J Pediatr Orthop.

[16] Carroll K, Coleman S, Stevens P (1997). Coxa vara: surgical outcomes of valgust osteotomies. J Pediatr Orthop.

[17] Desai SS, Johnson LO (1993). Long-term results of valgus osteotomy for congenital coxa vara. Orthop Relat Res.

[18] Widmann RF, Hresko T, Kasser JR, Millis MB (2001). Wagner multiple K-wire osteosynthesis to correct coxa vara in the young child: experience with a versatile ‘tailor-made’ high angle blade plate equivalent. J Pediatr Orthop.

[19] Sabharwal S, Mittal R, Cox G (2005). Percutaneous triplanar femoral osteotomy correction for developmental coxa vara. J Pediatr Orthop.

[20] Weighill FJ (1976). The treatment of developmental coxa vara by abduction subtrochanteric and intertrochanteric femoral osteotomy with special reference to the role of adductor tenotomy. Clin Orthop.

[21] Mooney JF (2012). Use of temporary external fixation to generate a “customized” osteotomy of proximal femur in pediatric patients. J Surg Orthop Adv.

[22] Skaggs DL, DuBois B, Kay RM, Hale JM, Tolo VT (2000). A simplified valgus osteotomy of the proximal femur in children. J Pediatr Orthop.

[23] Raney EM, Grogan DP, Hurley ME, Ogden JA (2002). The role of proximal femoral valgus osteotomy in Legg-Calvé-Perthes disease. Orthopedics.

[24] Yang SH, Huang SC (1997). Valgus osteotomy for congenital coxa vara. J Formos Med Assoc.

[25] Cordes S, Dickens DRV, Cole WG (1991). Correction of coxa vara in childhood. The use of Pauwels’s y-shaped osteotomy. J Bone Joint Surg Br.

[26] Shim JS, Kim HAT, Mubarak Sj, Wenger DR (1997). Genu valgum in children with coxa vara resulting from hip disease. J Pediatr Orthop.

